# Relation Between Training Load and Recovery-Stress State in High-Performance Swimming

**DOI:** 10.3389/fphys.2018.00845

**Published:** 2018-07-05

**Authors:** Robert Collette, Michael Kellmann, Alexander Ferrauti, Tim Meyer, Mark Pfeiffer

**Affiliations:** ^1^Department Theory and Practice of Sports, Institute of Sport Science, Johannes Gutenberg University Mainz, Mainz, Germany; ^2^Unit of Sport Psychology, Faculty of Sport Science, Ruhr-University Bochum, Bochum, Germany; ^3^School of Human Movement and Nutrition Sciences, The University of Queensland, Brisbane, QLD, Australia; ^4^Department of Training and Exercise Science, Faculty of Sport Science, Ruhr-University Bochum, Bochum, Germany; ^5^Institute of Sports and Preventive Medicine, Saarland University, Saarbrücken, Germany

**Keywords:** monitoring, training, recovery-stress state, internal load, session RPE, ACWR, time series analysis, individual case

## Abstract

**Background:** The relation between training load, especially internal load, and the recovery-stress state is of central importance for avoiding negative adaptations in high-performance sports like swimming. The aim of this study was to analyze the individual time-delayed linear effect relationship between training load and recovery-stress state with single case time series methods and to monitor the acute recovery-stress state of high-performance swimmers in an economical and multidimensional manner over a macro cycle. The Acute Recovery and Stress Scale (ARSS) was used for daily monitoring of the recovery-stress state. The methods session-RPE (sRPE) and acute:chronic workload-ratio (ACWR) were used to compare different methods for quantifying the internal training load with regard to their interrelationship with the recovery-stress state.

**Methods:** Internal load and recovery-stress state of five highly trained female swimmers [with a training frequency of 13.6 ± 0.8 sessions per week and specializing in sprint (50 and 100 m), middle-distance (200 and 400 m), or long distance (800 and 1,500 m) events] were daily documented over 17 weeks. Two different types of sRPE were applied: RPE^∗^duration (sRPE^h^) and RPE^∗^volume (sRPE^km^). Subsequently, we calculated the ratios ACWR^h^ and ACWR^km^ (sRPE last week: 4-week exponentially weighted moving average). The recovery-stress state was measured by using the ARSS, consisting of eight scales, four of which are related to recovery [Physical Performance Capability (PPC), Mental Performance Capability (MPC), Emotional Balance (EB), Overall Recovery (OR)], and four to stress [Muscular Stress (MS), Lack of Activation (LA), Negative Emotional State (NES), Overall Stress (OS)]. To examine the relation between training load and recovery-stress state a cross correlation (CCC) was conducted with sRPE^h^, sRPE^km^, ACWR^h^, and ACWR^km^ as lead and the eight ARSS-scales as lag variables.

**Results:** A large variation of training load can be observed in the individual week-to-week fluctuations whereby the single fluctuations can significantly differ from the overall mean of the group. The range also shows that the CCC individually reaches values above 0.3, especially with sRPE^km^ as lead variable. Overall, there is a large range with significant differences between the recovery and stress dimensions of the ARSS and between the training load methods, with sRPE^km^ having the largest span (*Range* = 1.16). High inter-individual differences between the athletes lie in strength and direction of the correlation | 0.66|≤ CCC ≥|-0.50|. The time delayed effects (lags 0–7) are highly individual, however, clear patterns can be observed.

**Conclusion:** The ARSS, especially the physical and overall-related scales (PPC, OR, MS, OS), is a suitable tool for monitoring the acute recovery-stress state in swimmers. MPC, EB, LA, and NES are less affected by training induced changes. Comparably high CCC and Ranges result from the four internal load methods, whereby sRPE, especially sRPE^km^, shows a stronger relation to recovery-stress state than ACWR. Based on these results and the individual differences in terms of time delay in training response, we recommend for swimming to use sRPE to monitor the internal training load and to use the ARSS, with a focus at the physical and overall-scales, to monitor the recovery-stress state.

## Introduction

Analyzing internal and external training loads has become a critical issue in elite sport practice and research. In this regard, monitoring the athletes internal training load is essential for understanding whether athletes are positively adapting to their training program. This implements an understanding of the individual’s responses to training, assessing fatigue and the associated need for recovery, in order to minimize the risk of non-functional overreaching, injury and illness (Bourdon et al., 2017; Kellmann and Beckmann, 2018; Kellmann et al., 2018). In high-performance sports like swimming an individual monitoring of the training load and the recovery-stress state for the prevention of negative adaptations is recommended (Foster et al., 1999; Smith, 2003; Lambert and Mujika, 2013; Collette, 2016; Soligard et al., 2016; Crowcroft et al., 2017).

Referring to Bourdon et al. (2017) the measures of *training load* can be categorized as either internal or external, where external training loads are objective measures of the work performed by the athlete (e.g., speed, acceleration, volume, …). On the other hand, internal training load is defined as the relative physiological and psychological stressors imposed on the athlete during training or competition. Various methods for measuring internal load exist, such as rating of perceived exertion (RPE), session rating of perceived exertion (sRPE), training impulse (TRIMP), heart-rate indices, blood lactate, oxygen uptake and/or psychological scales and questionnaires (Bourdon et al., 2017). At present, especially the ‘sRPE’ (Foster et al., 1999) as well as the ‘acute:chronic-workload ratio’ (ACWR) (Gabbett, 2016; Hulin et al., 2016) methods are being discussed, whereas sRPE has been extensively investigated and seems to be a valid tool for measuring internal training load in a variety of sport (Foster et al., 2001; Herman et al., 2006; Seiler and Kjerland, 2006; Borresen and Lambert, 2008), especially in swimming (Wallace et al., 2008, 2009; Toubekis et al., 2013). Nagle et al. (2015) modified the method of Foster by using the volume (km) instead of the duration for the calculation. It is assumed that in endurance sports such as swimming, volume has a greater impact on the recovery-stress state than duration. A validation study in which both methods are compared is not yet available. The ACWR is a simplification of the fitness-fatigue model of Banister et al. (1975) and it was recently reported to provide valid information regarding injury risk in team sports (Hulin et al., 2016; Gabbett, 2016; Bowen et al., 2017; Murray et al., 2017b). It is therefore reasonable to conclude that ACWR also provides valid information regarding the impact of the internal load on *recovery-stress state.*

The recovery-stress state is based on the individual’s ability to utilize resources necessary for recovery in order to compensate stressful situations and activities (Nässi et al., 2017). Stress and recovery appear to be complex, intertwined processes that should be viewed from different perspectives such as time frames and/or contexts, and even multiple processes (Kenttä and Hassmén, 1998; Kellmann and Kallus, 2001; Kellmann and Beckmann, 2018). In high-performance sport self-report measures via questionnaires represent the most common form for monitoring the athlete’s recovery-stress state (Nässi et al., 2017) and for this purpose, several valid and reliable instruments are available, e.g., the Profile of Mood States (POMS) (McNair et al., 1971) or the Recovery-Stress-Questionnaire for Sport (RESTQ-Sport) (Kellmann and Kallus, 2001, 2016) are the most frequently used instruments (Saw et al., 2016, 2017). However, both instruments do not examine the acute recovery-stress state in a multidimensional manner. The main criterion for POMS is the predominantly negative orientation of the questionnaire (Martin et al., 2000; Ziemainz and Peters, 2010) and that it has been developed for the assessment of mood (Hitzschke et al., 2016). However, recovery-stress state and mood are regarded as separate psychological constructs (Mäestu et al., 2005). The RESTQ-Sport includes 76 items and is therefore not suitable for a weekly or daily use (Kellmann, 2000). Additionally, recovery and stress state are evaluated over the last 3 days and therefore the questionnaire does not indicate the acute (‘here right now’) condition of the athlete (Kölling et al., 2015). Meeusen et al. (2013) point out that for effective load monitoring a shorter questionnaire or instrument is needed which responds sensitively to the current state of recovery and stress. As a result, Kellmann et al. (2016) developed the Acute Recovery and Stress Scale (ARSS) to assess and monitor the acute multidimensional recovery and stress state, that considers not only the physical, but also the emotional and psychological recovery or stress. The ARSS can be applied on a daily basis for training monitoring in elite sports (Hitzschke et al., 2017). Several laboratory and field studies in swimming (Collette, 2016), cycling (Hammes et al., 2016), rowing (Kölling et al., 2016), tennis (Wiewelhove et al., 2016), football (Pelka et al., 2017) or strength and high-intensity interval training (Raeder et al., 2016) showed the practicability and suitability to changes of the training stimuli of the ARSS. These studies showed that daily changes as well as indications for a general trend of the recovery-stress state in different training phases will be displayed.

Several studies have investigated the relationship between training load and well-being or recovery-stress state, with an increase in ‘stress’ and reduction in ‘recovery’ after intensive training, respectively, higher training load in comparison to normal training load. In addition, a reduction in stress scales and increase in recovery scales were also observed following a taper phase. Furthermore, the results show a high variability and indicate a high degree of individuality (Morgan et al., 1987; Berglund and Safstrom, 1994; Adams and Kirkby, 2001; O’Connor and Puetz, 2005; Coutts et al., 2007; Kellmann, 2010; Bresciani et al., 2011; Brink et al., 2012; Laux et al., 2015).

In high-performance sport, there is a high degree of individuality in terms of training loads and training adaptation (Collette, 2016; Julian et al., 2017). In addition, the recovery-stress structure is characterized by high individuality (Bouchard and Rankinen, 2001; Hautala et al., 2006; Hecksteden et al., 2015, 2017; Schimpchen et al., 2017).

However, the above-mentioned studies show a number of shortcomings: (a) mostly the focus was to compare group values, no single case study performed before; (b) typically pre–post study design was applied so that the process cannot be observed; (c) the studies usually comprise short periods or specific training phases (e.g., taper-phase, training camps); (d) the recovery-stress state was evaluated over a time period (e.g., over 3-days with the RESTQ-Sport), only one-dimensionally (e.g., RPE) or the mood is measured (e.g., POMS).

Considering these critical points, the aim of the present study was

(a) to analyze the individual time-delayed linear effect relationship between training load and recovery-stress state with single case time series methods (bivariate cross-correlations),
(b) to monitor the acute recovery-stress state of high-performance swimmers in an economical and multidimensional manner over a long period or different training periods (macro cycle)
(c) to compare different methods for quantifying the internal training load with regard to their interrelationship with the recovery-stress state,
(d) to detect differences in the relationship between internal load and the two states ‘recovery’ and ‘stress’ determined by using the ARSS.

## Materials and Methods

### Participants

Five female high-performance swimmers (S1–S5, mean ±*SD*: age: 21 ± 2.8 years, body mass: 60.1 ± 6.5 kg, height: 1.72 ± 0.1 m, best Fédération Internationale de Natation (2014) points in main event as percentage of world record 72.8 ± 7.9%) monitored daily over 17 weeks. All participants were well-trained athletes, accustomed to a training frequency of more than thirteen sessions per week (13.6 ± 0.8), including pool and athletic sessions. Specialized in sprint (50 and 100 m), middle-distance (200 and 400 m) or long distance (800 and 1,500 m) events.

The study received approval from the Ruhr-Universität Bochum, Faculty of Psychology, Ethics Committee. All participants gave their written informed consent to participate in the study which was conducted in accordance with the Declaration of Helsinki.

### Design

Athletes were monitored over a 17-week macrocycle (tw) which included different mesocycles and periodization phases, as well as one or two main competitions [German Championships (tw 9)/German Youth Championships (tw 16)]. Within these macrocycle a 16 days training camp was included at end of week three. The recovery-stress state was recorded every morning using the ARSS. Additionally, internal training load using sRPE was documented after every training session. In order to provide as much error-free documentation as possible, the training load was recorded by both the athletes themselves and the coach. The athletes were instructed to complete the ARSS questionnaire every morning before the first training session. The questionnaire was provided on an online platform^1^ and was filled out using the athlete’s smartphone or tablet. The compliance was high with data only missing for S1, S2 (1 day each/0.8%) and S5 (6 days/5.0%).

**FIGURE 1:**
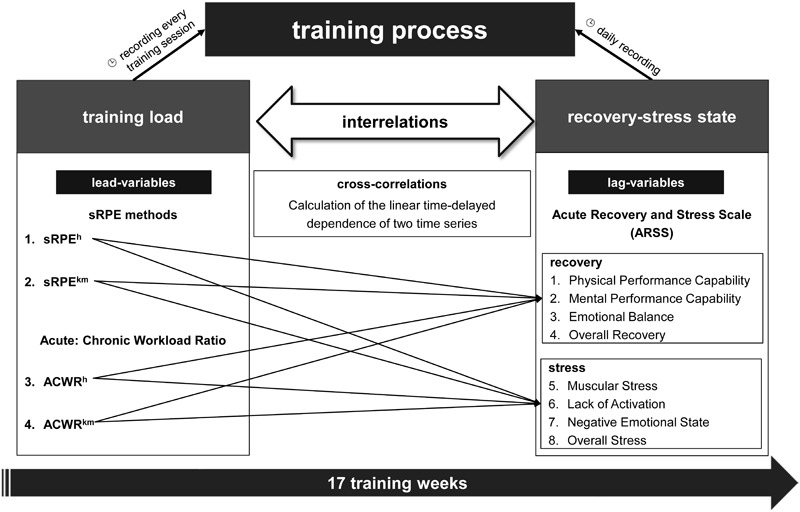
Systematic overview of the study design and the collected or calculated parameters. Cross-correlations were calculated for every of the four lead-variables with every scale of the ARSS as lag-variable. sRPE^h^ = RPE^∗^duration (min); sRPE^km^ = RPE^∗^volume (km); ACWR^h^ = sRPE^h^ last week/sRPE^h^ 4-week exponentially weighted moving average; ACWR^km^ = sRPE^km^ last week/sRPE^km^ 4-week exponentially weighted moving average.

For calculations the following parameters were individually collected: duration of every training session (min); volume (km); sRPE; ARSS. **Figure 1** shows a systematic overview of the study design as well as the collected or calculated parameters.

### Training Load

Two different methods of sRPE were used to quantify the internal training load and based on the sRPE values the ‘acute:chronic workload ratios’ (ACWR) were calculated (**Table 1**).

**Table 1:** Overview of the various calculation formulas for the methods of training load quantification.

Method	Calculation formula	Unit
sRPE^h^	= RPE^∗^duration (min)	AU
ACWR^h^	= sRPE^h^ last week/sRPE^h^ 4-week exponentially weighted moving average	AU
sRPE^km^	= RPE^∗^volume (km)	AU
ACWR^km^	= sRPE^km^ last week/sRPE^h^ 4-week exponentially weighted moving average	AU

*sRPE, session rating of perceived exertion; ACWR, acute:chronic workload ratio; AU, arbitrary units.*

#### Session RPE

As described by Foster et al. (2001) within 30 min after every training session participants were given standard instructions for overall RPE and were asked to report based on the degree of whole body heaviness and strain experienced during the exercise task using a 11-point scale (based on the CR-10 scale by Borg, 1982), with 0 and 10 corresponding to ‘rest’ and ‘maximal,’ respectively.

For the first method, *sRPE^h^*, the training load was calculated by multiplying the 0–10 rating by the total session duration (in min) and expressed in arbitrary units (Foster et al., 2001). Weekly sRPE^h^ training load was calculated for each athlete individually by summing the sRPE^h^ training loads for all training sessions.

The second method, *sRPE^km^*, differs only in a modified calculation for the sRPE-method by using volume (in km) instead of duration (in min) (Nagle et al., 2015). Weekly sRPE^km^ training load scores were calculated for each athlete individually by summing the sRPE^km^ training loads for all training sessions. To consider dryland workouts the duration of the session was converted to volume based on Mujika et al. (1996) (60-min dryland session equals 2 km of swimming).

#### Acute:Chronic Workload Ratio

The training load (sRPE) of 1 week is defined as the acute load and the chronic training load represent the exponentially weighted moving average (EWMA) of the load in the four previous weeks of training (Nielsen et al., 2014; Gabbett, 2016; Murray et al., 2017a).

As described by Williams et al. (2016) and Murray et al. (2017a), the EWMA is calculated as follows:

$${\text{EWMA}}_{\text{today}}\;\text{=}\;{\text{sRPE}}_{\text{today}}\times \;\lambda_{\text{a}}\text{+}((\text{1-}\lambda_{\text{a}})\times {\text{EWMA}}_{\text{yesterday}})$$

and λ_a_ iscalculatedas :

$$\lambda_{\text{q}}\;\text{=}\;\text{2/}(\text{N+1})$$

Where *N* is the time decay constant with 1 week (7 days) for acute and 4 weeks (28 days) for chronic workloads. For the ratio the *EWMA* ACWR value of acute workload was divided by the *EWMA* ACWR value of chronic workload. To begin the EWMA calculation, the first observation in the series is arbitrarily recorded as the first workload value in the series (Murray et al., 2017a).

Comparing the acute training load to the chronic training load as a ratio provides an index of athlete preparedness and fatigue (Gabbett, 2016). The ACWR was divided into the following ranges: very low ≤ 0.49, low 0.5–0.99, moderate 1.0–1.49, high 1.50–1.99, and very high 2.0 (Murray et al., 2017a). As such an ‘acute:chronic workload ratio’ between 0.8 and 1.3 was considered the ‘sweet spot,’ while ratios ≥ 1.5 represent the ‘danger zone’ with an increased risk of injury (Blanch and Gabbett, 2016; Gabbett, 2016). The ‘acute:chronic load ratio’ calculated with training loads based on the sRPE^h^- (*ACWR^h^*) and sRPE^km^-Method (*ACWR^km^*) is shown in **Table 1**.

### Acute Recovery and Stress Scale

The ARSS consists of a total of 32 adjectives describing the physical, emotional, mental, and general aspects of recovery and stress based on a 7-point Likert scale from 0 (does not apply at all) to 6 (fully applies) (Kellmann et al., 2016). The adjectives are summarized in eight scales, of which four are related to stress, and four to recovery. The recovery-related scales are: *Physical Performance Capability (PPC), Mental Performance Capability (MPC), Emotional Balance (EB)*, and *Overall Recovery (OR)*. The stress-related scales are: *Muscular Stress (MS), Lack of Activation (LA), Negative Emotional State (NES)*, and *Overall Stress (OS)* (Hitzschke et al., 2017). All scales of the German ARSS showed satisfactory internal consistency (range between α = 0.84 and α = 0.96) and a good model fit for both the recovery (RMSEA = 0.07, CFI = 0.97, SRMR = 0.04) and stress (RMSEA = 0.09, CFI = 0.94, SRMR = 0.05) factors (Kellmann et al., 2016). Nässi et al. (2017) reported the psychometric properties of the English version of the ARSS.

### Statistical Analyses

**FIGURE 2:**
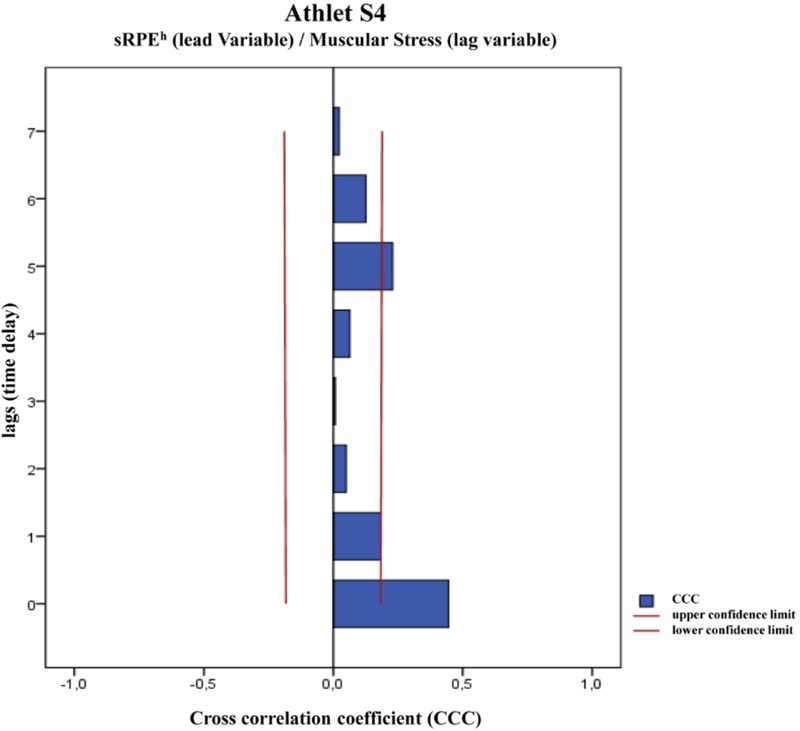
Example of a cross-correlation plot for Athlete S4 with sRPE^h^ as lead-variable and the ARSS-Dimension *Emotional Balance* as lag-variable.

The time-series analysis is designed to investigate the time-lagged effects of several variables, in which the correlations between the training loads and the recovery-stress state are investigated using bivariate cross-correlations. In other words, the linear dependence on the time delay of two time series is calculated here (Schmitz, 2000). For this purpose, two time series are shifted against each other by π-times, resulting in a lead variable and a lag variable. The direction of the displacement, e.g., the variable which is the lead and the lag variable (Schmitz, 1996) is important. As lead variables, the sRPE and ACWR values are set in this study in order to examine the time-delayed effects of the training on the recovery-stress state in terms of the eight scales of the ARSS as lag variables. This means that for every athlete 32 cross-correlations (**Figure 1**) with *n* = 119 data points (days) were calculated. A significant correlation can be interpreted in the sense of a co-determination of the lag variable by the lead variable (Schmitz, 2000). According to Maiwald and Rogge (2005), significant cross-correlations are found for physiological variables and ordinally scaled self-estimates when their absolute value is greater than CCC ≥ 0.2. Due to the method of cross-correlation, only the maximum significant cross-correlation coefficients (max. CCC) with the associated time delay (lag) are of interest for further evaluation. In order to avoid the possibility of false inconsistencies, a ‘pre-whitening’ (data transformation into ‘White Noise’) of the time series is deliberately dispensed with, which is why the strengths of the cross-correlation coefficients can only be interpreted with extreme caution, especially in the case of interindividual comparisons (Schmitz, 1996). The IBM© SPSS© Statistics 23 software package was used to perform the complex statistical calculations. The cross-correlation logs have been calculated for seven lags (7 days) and show the corresponding 95% confidence intervals (e.g., **Figure 2**). Since the time-series analyzes are very sensitive to erroneous data, the few missing data are estimated. The estimated value was determined by calculating the mean value 2 days before and after the data gap. The calculation of the mean values of the cross-correlation coefficients implemented the Fischer-Z transformations (Bortz and Schuster, 2010).

## Results

### Training Load

**FIGURE 3:**
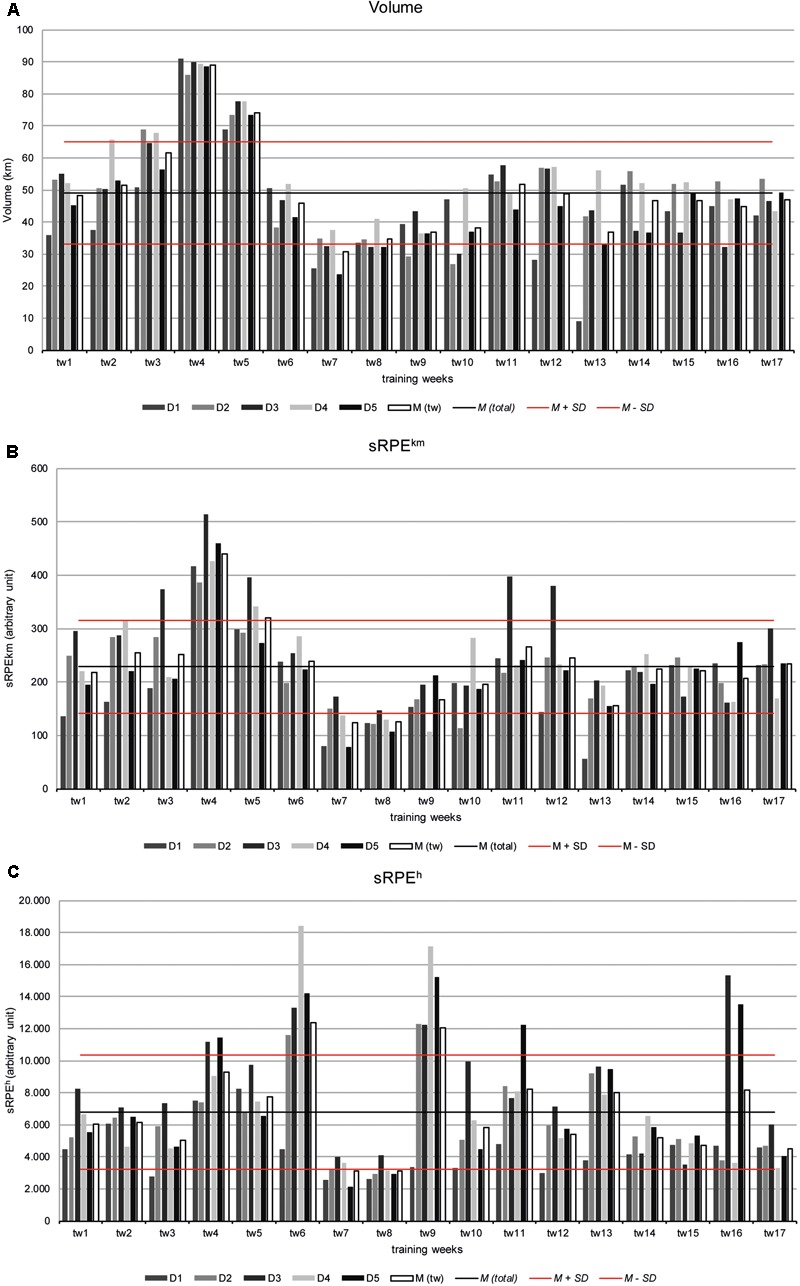
Training load **(A)** volume (km) **(B)** sRPE^km^ and **(C)** sRPE^h^ over 17 training weeks of the athletes S1–S5 as well as the mean values [*M* (tw)]. To estimate the changes from week to week, the total mean value [*M* (total)] as well as plus/minus a standard deviation (*M ± SD*) for reference are indicated.

Total mean training volume was 833.7 ± 14.1 km with a mean maximum of 89.0 km in tw 4 and a minimum of 30.8 km in tw 7 (**Figure 3A**).

Total mean *sRPE^km^*-load was 3888.1 ± 72.4 au, with a maximum (440.5 au) in tw 4 and a minimum (123.6 au) in tw 7 (**Figure 3B**).

The *sRPE^h^* values also show a similar distribution as the sRPE^km^ values, with the peak loads striking in particular during the training weeks with competitions (tw 6, tw 9, tw 16). Mean sRPE^h^-training load was 114996 ± 2630 au (**Figure 3C**).

Overall, a large variation can be observed in the individual week-to-week fluctuations whereby the single fluctuations can significantly differ from the overall mean plus/minus the standard deviation of the group (**Figures 3A–C**).

Thus, the individual maximum for volume (91.0 km in tw 4) and sRPE^h^ (2110 au in tw 7) can be seen for athlete S1, while athlete S3 has the maximum for sRPE^km^ (18390 au in tw 6). The individual minimum for volume (9.1 km in tw 13) and sRPE^km^ (55.3 au in tw 13) has also athlete S1, while S5 shows the minimum for sRPE^h^ (2110 au in tw 7) (**Figures 3A–C**).

**FIGURE 4:**
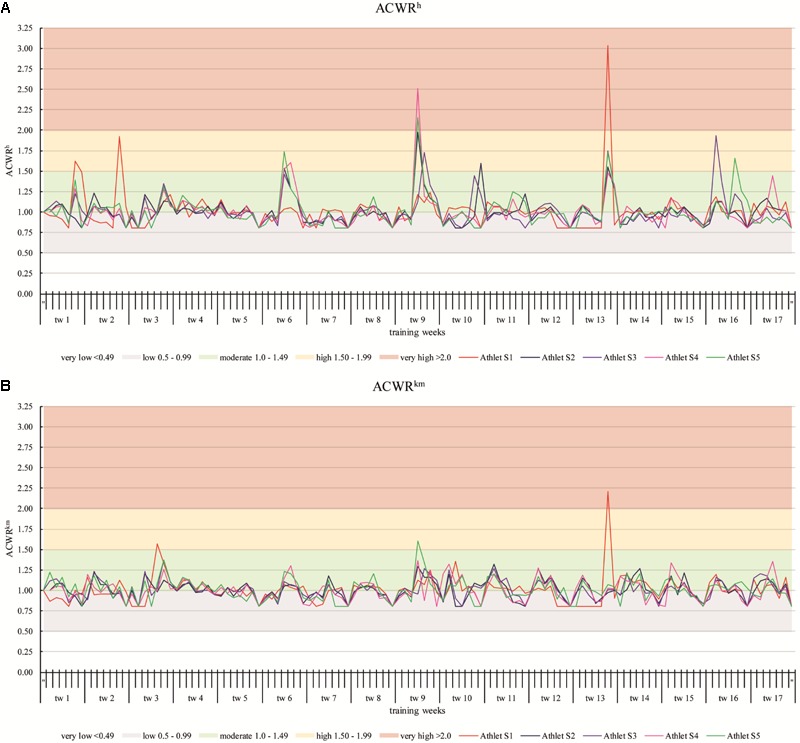
The acute:chronic workload-ratio over 17 training weeks of the athletes S1–S5 based on the sRPE^h^- **(A)** and the sRPE^km^-values **(B)**.

**Figure 4** shows the ‘ACWR’ based on the sRPE^h^- (**Figure 4A**) and the sRPE^km^-values (**Figure 4B**). Only the *ACWR^h^* curves of Athletes S1, S4, and S5 are in the ‘very high’ (>2.0) range and these are only short-term peaks over a day. Except for S1 all athletes have a peak in the range of 1.5–1.99 (high) or >2.0 in training week 9 in which the German Championships took place. In comparison, the values in the training camp (end of tw 3 to tw 5) are all in moderate range (1.0–1.49). In the *ACWR^km^* curves only S1 shows a peak in the very high range and this, as with *ACWR^h^*, in training week 13. In addition, peaks in the high range can only be seen S1 and S5. Overall, the curves of *ACWR^km^* are smoother compared to *ACWR^h^* and predominantly in the low (0.5–0.99) to moderate (1.0–1.49) range.

#### Cross-Correlation

**FIGURE 5:**
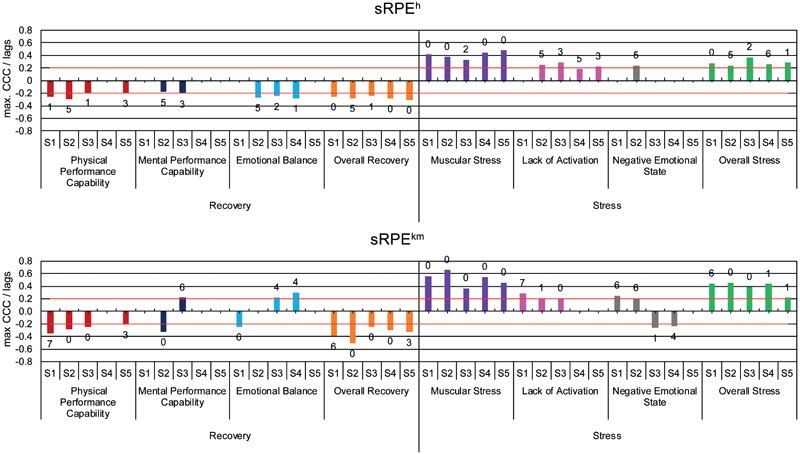
Maximum significant cross correlations (max CCC) between the dimensions of ARSS as lag variables and sRPE^h^ and sRPE^km^ as lead variables with the associated time delay (lags) for the athletes S1 - S5.

**FIGURE 6:**
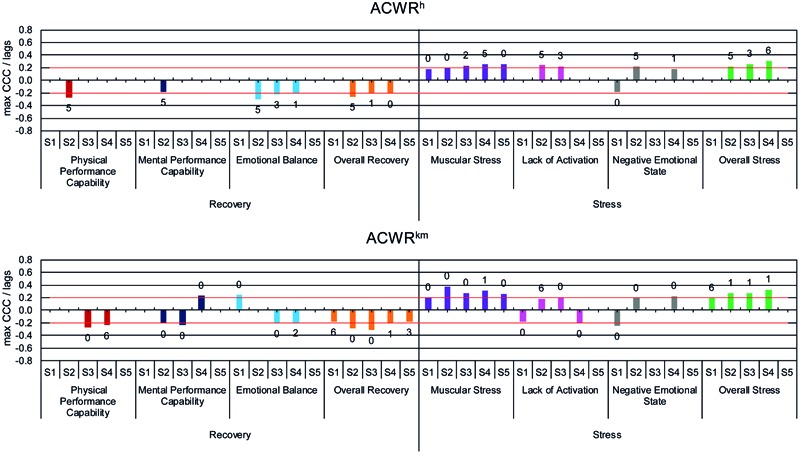
Maximum significant cross correlations (max CCC) between the dimensions of ARSS as lag variables and ACWR^h^ and ACWR^km^ as lead variables with the associated time delay (lags) for the athletes S1–S5.

Only the maximum significant CCC is considered for further analysis due to methodological reasons of the cross-correlation (see above). **Table 2** shows the mean (*MCCC*) and the ranges of the CCC between the sRPE and ACWR values as lead variables and the dimensions of the ARSS, separated into recovery and stress, as lag variables. The highest *MCCC* are observed for *MS* with sRPE^h^ (*MCCC* = 0.41), respectively, sRPE^km^ (*MCCC* = 0.51) and for *OS* with sRPE^km^ (*MCCC* = 0.39). The range in **Table 2** also shows that the CCC individually reaches values above 0.3 in other dimensions, especially with sRPE^km^ as lead variable. Overall, there is a large range with significant differences between the recovery and stress dimensions of the ARSS, and between the training load methods, with sRPE^km^ having the largest span of *Range* = 1.16. Furthermore, it is noticeable that for sRPE^km^, ACWR^km^, and ACWR^h^ for the dimensions *MPC, EB, LA*, and *NES* ranges from negative to positive CCC, and thus different effective directions are present. Therefore, **Figures 5, 6** show the level and effective direction of the individual CCC, as well as the time delay, based on their lags. Contrary directions of action show for athletes S1, S3, and S4 but for different lead variables and dimensions, whereby only the mental and emotional dimensions (*MPC, EB, LA, NES*) are shown. S3 (*MPC* CCC = 0.22, *EB* CCC = 0.22, *NES* CCC = -0.26) and S4 (*EB* CCC = 0.30, *NES* CCC = -0.24) show for sRPE^km^ contrary effective directions, for ACWR^h^ only S1 (*NES* CCC = -0.19) and for ACWR^km^ S1 (*EB* CCC = 0.24, *LA* CCC = -0.18, *NES* CCC = -0.25), and S4 (*MPC* CCC = 0.23, *LA* CCC = -0.21). This also shows that there are more significant CCC values with the stress dimensions than with the recovery dimensions and in some cases, there are even considerably higher CCC.

**Table 2:** Mean (*MCCC*) and range of significant cross-correlations.

		sRPE^h^		sRPE^km^		ACWR^h^		ACWR^km^	
		*MCCC*	Range	*MCCC*	Range	*MCCC*	Range	*MCCC*	Range
*Recovery*	*PPC*	-0.23	(-0.18/-0.29)	-0.27	(-0.21/-0.35)	-0.27	(-0.27/-0.27)	-0.25	(-0.24/-0.27)
	*MPC*	-0.20	(-0.18/-0.21)	-0.06	(0.22/-0.32)	-0.18	(-0.18/-0.18)	-0.21	(0.23/-0.24)
	*EB*	-0.26	(-0.25/-0.28)	0.26	(0.30/-0.24)	-0.25	(-0.22/-0.30)	-0.20	(0.24/-0.20)
	*OR*	-0.27	(-0.25/-0.30)	**-0.36**	(-0.24/-0.50)	-0.22	(-0.23/-0.41)	-0.27	(-0.18/-0.31)
*Stress*	*MS*	**0.41**	(0.48/0.33)	**0.52**	(0.66/0.37)	0.22	(0.26/0.18)	0.29	(0.38/0.20)
	*LA*	0.24	(0.18/0.29)	0.21	(0.29/0.21)	0.23	(0.24/0.22)	0.20	(0.21/-0.21)
	*NES*	0.24	(0.24/0.24)	0.21	(0.25/-0.26)	0.22	(0.22/-0.19)	0.19	(0.23/-0.25)
	*OS*	0.29	(0.37/0.24)	**0.39**	(0.46/0.21)	0.26	(0.31/0.22)	0.29	(0.33/0.19)

*PPC, Physical Performance Capability, MPC, Mental Performance Capability, EB, Emotional Balance, OR, Overall Recovery, MS, Muscular Stress, LA, Lack of Activation, NES, Negative Emotional State, OS, Overall Stress. MCCC > 0.3 highlighted bold.*

In addition to the magnitude of the relationship between training load and recovery-stress state, from a training control perspective the time-delay of the relation is of interest. Our findings show high inter-individual as well as intra-individual differences of the time delayed effects, concerning the athletes and each dimension of the ARSS with lag 0 to 6 for sRPE^h^, ACWR^km^, ACWR^h^, and 0 to 7 for sRPE^km^.

**FIGURE 7:**
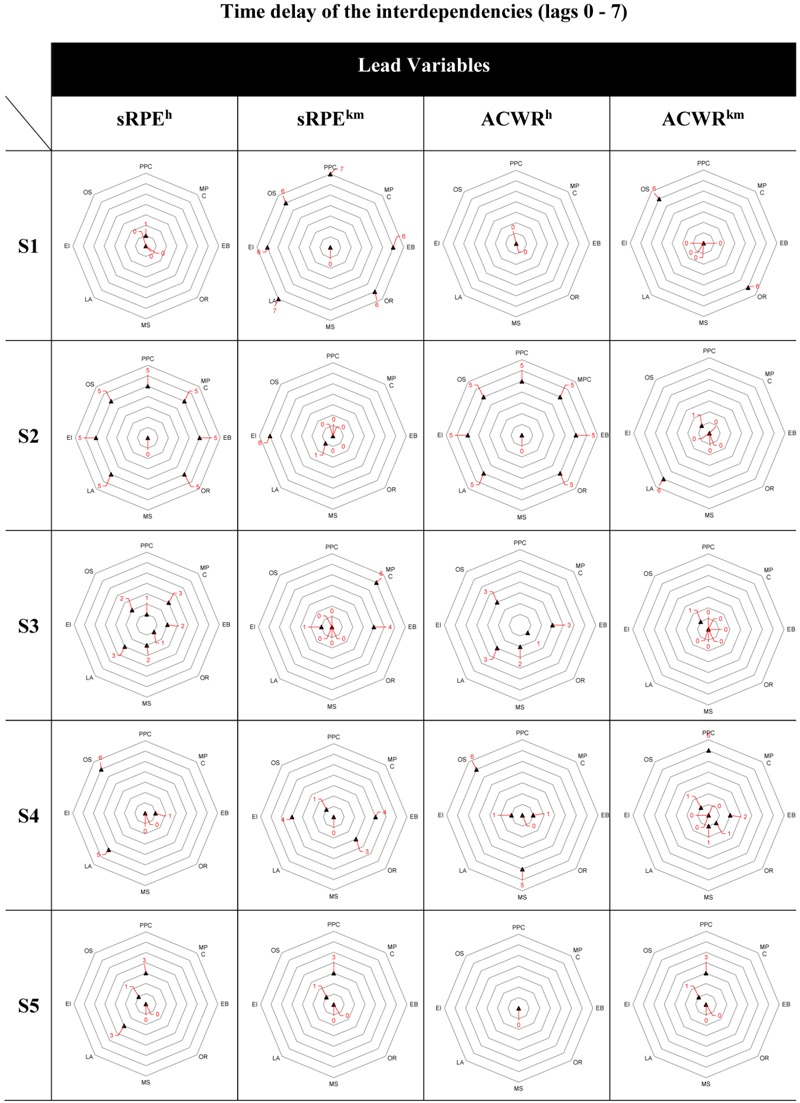
Individual profile display of the time delays (lag 0–7) for the examined athletes S1–S5 for the recovery and stress dimensions of the ARSS with sRPE^h^, sRPE^km^, ACWR^h^, and ACWR^km^ as lead variables on the basis of net diagrams. PPC, Physical Performance Capability; MPC, Mental Performance Capability; EB, Emotional Balance; OR, Overall Recovery; MS, Muscular Stress; LA, Lack of Activation; NES, Negative Emotional State; OS, Overall Stress.

To make the time-delayed effects comparable with each other, an individual profile using a network diagram was created for each athlete and for all four lead variables. **Figure 7** shows the individual profiles in terms of time-delayed interaction for the recovery (*PPC, MPC, EB, OR)* and stress dimensions (*MS, LA, NES, OS)* of the ARSS with sRPE^h^, sRPE^km^, ACWR^h^, and ACWR^km^ as lead variables. If sRPE^h^ and ACWR^h^ are not taken into account only once for S3 and sRPE^km^ for S4, three basic tread patterns can be distinguished. The athletes S1 (sRPE^h^, ACWR^h^), S3 (ACWR^km^), and S5 (sRPE^h^, sRPE^km^, ACWR^h^, ACWR^km^) react very quickly or directly with lag 0 or lag 1 (or a single maximum lag 3 for S5) in dimensions where significant correlations on the individual training load exist (Profile 1). Profile 2 shows only a small deviation; one or two dimensions react with a significantly larger time delay (lag 4 to 6). For S2 (*NES, LA*) and S3 (*MPC, EB*) these are only mental or emotional-related scales, for S1 only the overall scales (*OR, OS*) and for S4 (*PPC, MS, LA, OS*) both occurs. Profile 3 is distinctly different from the other two profiles, as all dimensions except for *MS* (lag 0) react explicitly later with lag 5 to 7. It can be noticed that for all athletes and all load methods only for *MS*, the time-delayed effect is always between 0 and 2. The only exception here is athlete S4 for ACWR^h^ with lag 5.

## Discussion

The time-delayed linear effect relationship, which has been calculated according to the time series analysis using bivariate cross-correlations between the individual training load and the dimensions of the ARSS, confirm the theoretical assumption that the interactions between training load and the recovery-stress state is characterized with high inter- and intra-individual differences. It also reaffirms the idea that recovery and stress should be explored using a multi-level approach that takes into account psychological, emotional, cognitive, and social aspects both individually and collectively (Kellmann, 2002b). High inter-individual differences between the athletes lie in strength and direction of the correlation |0.66|≤ CCC ≥|-0.50| as well as in the time delays from lag 0 to lag 7.

Low values in the stress dimensions and high values in the recovery dimensions are generally defined as positive and vice versa as negative. However, in this context terms like “good – bad” and/or “positive – negative” should be used with care (Kellmann, 2010). The results show that the linear directions of action between the training loads and the individual recovery- and stress-related scales are in contrast to this assumption for three athletes (S1, S3, and S4). High training loads lead to higher values in the recovery dimensions and lower values in the stress dimensions, however, in different dimensions and with different lead variables. Another aspect is, that the contradictory directions of action exist only for the mental- (*Mental Performance Capacity* and *Lack of Aviation*) and emotional-related scales (*Emotional Balance* and *Negative Emotional State*). In a study of elite rowers Kellmann (2002a) also concluded, that at high training loads, individual recovery parameters, in this case the items *physical recovery* and *fun* of the ‘Recovery Cue’ (Kellmann et al., 2002) can be quite lead to higher values. These results illustrate that the relationship between training load and recovery-stress state cannot be easily generalized, because the interdependencies seem to be too complex and highly individual. A possible explanatory approach can be provided via the ‘Individual Zones of Optimal Functioning’ model of Hanin (Hanin, 1980, 2002; Salminen et al., 1995; Hanin and Hanina, 2009), which supports the assumption of an individual area or zone in which training loads are more likely to have positive effects on the recovery-stress state. The optimal level of training load does not always occur at the midpoint of the continuum but rather varies from individual to individual. That is, some athletes have a zone of optimal functioning at the lower end of the continuum, some in the midrange, and others in the upper end (Weinberg and Gould, 2007). In addition, the optimal level is most likely not a specific point but rather includes a particular individual range. Another step would be to analyze the relationship of performance against an appropriate modeling to determine individual profiles or individual optimal zones for the dimensions of the ARSS on this basis. In accordance with this, some authors proposed to conduct repeated measurements to establish an individual baseline from which changes can be determined (Saw et al., 2016, 2017; Hecksteden et al., 2017; Hitzschke et al., 2017).

The results support the sensitivity of the ARSS, as the overall and physical-related scales and items showed largest changes in response to the physical stress stimulus. Furthermore, the results underline recent indications from Kölling et al. (2015) and Hitzschke et al. (2017) that the subjective ratings of the physical and overall-related scales *Physical Performance Capacity, Overall Recovery, Muscular Stress* and *Overall Stress* respond on acute load. Nevertheless, mental- and emotional-related scales were affected as well, however, with low cross-correlations. This indicates that these dimensions might be more affected by non-training-induced stressors or factors. For the analysis of the relationship between training load and recovery-stress state the mental- and emotional-related scales seem not to be suitable for this sample.

The difference in time-delay between the sRPE and the ACWR method was expected due to the calculation method of the ACWR using a ratio 1:4 by means of EWMA. The influence of the method for recording the internal load on time delay is clearly visible. Thus, it might be suspected that the ACWR might not be suitable to investigate the time-delayed interdependencies of load and recovery-stress state. However, ACWR still provides a simple and practical method for monitoring load, especially with regards to the control of load intensity over longer periods of time (Hulin et al., 2014; Blanch and Gabbett, 2016; Gabbett et al., 2016; Soligard et al., 2016).

sRPE^h^ and sRPE^km^ appear to be the more suitable methods for monitoring the interdependencies between load and recovery-stress state, although significant differences were observed. For example, S1 equals profile 1 for sRPE^h^ and profile 3 for sRPE^km^, whereas the inverse applies to S2 (sRPE^h^ profile 3 and sRPE^km^ profile 1). Also in terms of the number of CCC and the amount of significant CCC, the sRPE-methods seem to be better suited for monitoring than the ACWR-methods. The results indicate that the more suitable method for swimming is sRPE^km^, which suggests that in swimming the influence of the volume on the perceived exertion is greater than the training time. This has to be further investigated. In particular, because the sRPE^km^ method was used by Nagle et al. (2015) as a modification of Foster’s sRPE method (Foster et al., 2001) without previously performing any studies on the comparability of the methods.

### Limitations

One limitation of the current study can be seen in the small sample size and that only female athletes were examined. To find a sufficient number of highly trained athletes with high compliance for studies is a general problem. To reach the target position beyond the individual case to a typology or group statements may with this group size only pointing the way for further investigation. From a sports practical point of view, it is clear that single case analysis is sensible and necessary for athlete monitoring. On the other hand, for a scientific generalization based on a group statistic, the sample is too small. So further single case studies are required to investigate whether different types of athletes can be differentiated. Even the decision to perform no ‘pre-whitening’ for the time series analysis that can lead to ‘apparent correlations’ in the presence of serial dependence, is to question critically. However, the procedures for how to perform this filtering are particularly controversial for studies with psychological parameters and are in part rejected, especially as there is a risk that the problem will be reversed and ‘fake independence’ will be present. In addition, it may be criticized that, for the analysis of the relationships between training load and recovery-stress state, the time-delayed effects were examined linear by bivariate cross-correlations and not by multivariate non-linear models. For future investigations, suitable non-linear models should be used or developed. Furthermore, an individual profile or individual optimum recovery-stress zone should be defined, relating to corresponding performance data.

## Conclusion

Based on our data we conclude that the ARSS, especially the physical and overall-related scales (*PPC, OR, MS, OS*), is a suitable tool for monitoring the acute recovery-stress state in swimmers. Due to its sensitivity to training loads, the individual time-delayed correlations between internal load and recovery-stress state was demonstrated. These interrelationships are considered in relation with CCC > 0.2 to be significant and in many cases as high, e.g., for *Muscular Stress* with sRPE^km^ for athlete S2 (CCC = 0.66).

For *Physical Performance Capability, Overall Recovery, Muscular Stress* and *Overall Stress* the effectiveness of training load on the recovery-stress state is in line with the theory. *Mental Performance Capability, Emotional Balance, Lack of Activation*, and *Negative Emotional State* appear to be less training-induced. Comparably high CCC and Ranges result from the four internal load methods, whereby sRPE, especially sRPE^km^, shows a stronger relation to recovery-stress state than ACWR. Based on these results and the individual differences in terms of time delay in training response, we recommend performing intra individual evaluations as well as repeated measurements to establish an individual baseline from which changes can be determined in order to enable a training optimization through individual athletes monitoring. For swimming we also recommend using session RPE, especially sRPE^km^, to monitor the internal training load and to use the ARSS with focus on physical and overall-scales to monitor the recovery-stress state.

## Author Contributions

RC planned and designed the study, conducted measurements, analyzed the data, and prepared the manuscript. MP analyzed the data and edited the manuscript. MK, AF, and TM edited the manuscript.

## Conflict of Interest Statement

The authors declare that the research was conducted in the absence of any commercial or financial relationships that could be construed as a potential conflict of interest.

## Footnotes

1 www.soscisurvey.de

## References

[B1] Adams J. Kirkby R. (2001). Exercise dependence and overtraining. The physiological and psychological consequences of excessive exercise. *Sports Med. Train. Rehabil.* 10 199–222. 10.1080/10578310210395

[B2] Banister E. W. Calvert I. W. Savage M. V. Bach I. M. (1975). A system model of training for athletic performance. *Aust. J. Sci. Med. Sport* 7 57–61.

[B3] Berglund B. Safstrom H. (1994). Psychological monitoring and modulation of training load of world-class canoeists. *Med. Sci. Sports Exerc.* 26 1036–1040. 10.1249/00005768-199408000-00016 7968421

[B4] Blanch P. Gabbett T. J. (2016). Has the athlete trained enough to return to play safely? The acute:chronic workload ratio permits clinicians to quantify a player’s risk of subsequent injury. *Br. J. Sports Med.* 50 471–475. 10.1136/bjsports-2015-095445 26701923

[B5] Borg G. (1982). Psychophysical bases of perceived exertion. *Med. Sci. Sports Exerc.* 14 377–381. 10.1249/00005768-198205000-00012 7154893

[B6] Borresen J. Lambert M. I. (2008). Quantifying training load: a comparison of subjective and objective methods. *Int. J. Sports Physiol. Perform.* 3 16–30. 10.1123/ijspp.3.1.16 19193951

[B7] Bortz J. Schuster C. (2010). *Statistik für Human- und Sozialwissenschaftler [Statistics for Human and Social Scientists]. Vollständig Überarbeitete und Erweiterte Auflage* Vol. 7 Berlin: Springer-Verlag 10.1007/978-3-642-12770-0

[B8] Bouchard C. Rankinen T. (2001). Individual differences in response to regular physical activity. *Med. Sci. Sports Exerc.* 33 S446–S451. 10.1097/00005768-200106001-00013 11427769

[B9] Bourdon P. C. Cardinale M. Murray A. Gastin P. Kellmann M. Varley M. C. (2017). Monitoring athlete training loads. Consensus statement. *Int. J. Sports Physiol. Perform.* 12(Suppl. 2) S2161–S2170. 10.1123/IJSPP.2017-0208 28463642

[B10] Bowen L. Gross A. S. Gimpel M. Li F.-X. (2017). Accumulated workloads and the acute:chronic workload ratio relate to injury risk in elite youth football players. *Br. J. Sports Med.* 51 452–459. 10.1136/bjsports-2015-095820 27450360 PMC5460663

[B11] Bresciani G. Cuevas M. J. Molinero O. Almar M. Suay F. Salvador A. (2011). Signs of overload after an intensified training. *Int. J. Sports Med.* 32 338–343. 10.1055/s-0031-1271764 21380974

[B12] Brink M. S. Visscher C. Coutts A. J. Lemmink K. A. P. M. (2012). Changes in perceived stress and recovery in overreached young elite soccer players. *Scand. J. Med. Sci. Sports* 22 285–292. 10.1111/j.1600-0838.2010.01237.x 21039901

[B13] Collette R. (2016). *Trainings- und Erholungsmonitoring im Leistungssport Schwimmen: [Monitoring Training and Recovery in Competitive Swimming]* Vol. 1. Hamburg: Kovac.

[B14] Coutts A. J. Wallace L. K. Slattery K. M. (2007). Monitoring changes in performance, physiology, biochemistry, and psychology during overreaching and recovery in triathletes. *Int. J. Sports Med.* 28 125–134. 10.1055/s-2006-924146 16835823

[B15] Crowcroft S. McCleave E. Slattery K. Coutts A. J. (2017). Assessing the measurement sensitivity and diagnostic characteristics of athlete-monitoring tools in national swimmers. *Int. J. Sports Physiol. Perform.* 12 S295–S2100. 10.1123/ijspp.2016-0406 27736255

[B16] Fédération Internationale de Natation (2014). *FINA Points Table.* Available at: http://www.fina.org/content/fina-points

[B17] Foster C. Florhaug J. A. Franklin J. Gottschall L. Hrovatin L. A. Parker S. (2001). A new approach to monitoring exercise training. *J. Strength Cond. Res.* 15 109–115. 11708692

[B18] Foster C. Snyder A. Welsh R. (1999). “Monitoring of training, warm up, and performance in athletes,” in *Overload, Performance Incompetence, and Regeneration in Sport* eds Lehmann M. Foster C. Gastmann U. Keizer H. Steinacker J. M. (New York, NY: Kluwer Academic) 43–51. 10.1007/978-0-585-34048-7_4

[B19] Gabbett T. J. (2016). The training-injury prevention paradox: should athletes be training smarter and harder? *Br. J. Sports Med.* 50 273–280. 10.1136/bjsports-2015-095788 26758673 PMC4789704

[B20] Gabbett T. J. Hulin B. T. Blanch P. Whiteley R. (2016). High training workloads alone do not cause sports injuries: how you get there is the real issue. *Br. J. Sports Med.* 50 444–445. 10.1136/bjsports-2015-095567 26795610

[B21] Hammes D. Skorski S. Schwindling S. Ferrauti A. Pfeiffer M. Kellmann M. (2016). Can the Lamberts and Lambert submaximal cycle test indicate fatigue and recovery in trained cyclists? *Int. J. Sports Physiol. Perform.* 11 328–336. 10.1123/ijspp.2015-0119 26263163

[B22] Hanin Y. (1980). “A study of anxiety in Sports,” in *Sport Psychology: An Analysis of Athlete Behavior* ed. Straub W. (Ithaca, NY: Mouvement Publications) 236–249.

[B23] Hanin Y. (2002). “Individually optimal recovery in sports: an application of the IZOF model,” in *Enhancing Recovery: Preventing Underperformance in Athletes* ed. Kellmann M. (Champaign, IL: Human Kinetics) 199–217.

[B24] Hanin Y. Hanina M. (2009). Optimization of performance in top-level athletes. An action-focused coping approach. *Int. J. Sports Sci. Coach.* 4 47–91. 10.1260/1747-9541.4.1.47

[B25] Hautala A. J. Kiviniemi A. M. Mäkikallio T. H. Kinnunen H. Nissilä S. Huikuri H. V. (2006). Individual differences in the responses to endurance and resistance training. *Eur. J. Appl. Physiol.* 96 535–542. 10.1007/s00421-005-0116-2 16369817

[B26] Hecksteden A. Kraushaar J. Scharhag-Rosenberger F. Theisen D. Senn S. Meyer T. (2015). Individual response to exercise training - a statistical perspective. *J. Appl. Physiol.* 118 1450–1459. 10.1152/japplphysiol.00714.2014 25663672

[B27] Hecksteden A. Pitsch W. Julian R. Pfeiffer M. Kellmann M. Ferrauti A. (2017). A new method to individualize monitoring of muscle recovery in athletes. *Int. J. Sports Physiol. Perform.* 12 1137–1142. 10.1123/ijspp.2016-0120 27967274

[B28] Herman L. Foster C. Maher M. A. Mikat R. P. Porcari J. P. (2006). Validity and reliability of the session RPE method for monitoring exercise training intensity. *S. Afr. J. Sports Med.* 18 14–17. 10.17159/2078-516X/2006/v18i1a247

[B29] Hitzschke B. Holst T. Ferrauti A. Meyer T. Pfeiffer M. Kellmann M. (2016). Entwicklung des Akutmaßes zur erfassung von erholung und beanspruchung im Sport. [Development of the acute recovery and stress scale]. *Diagnostica* 62 212–226. 10.1026/0012-1924/a000155

[B30] Hitzschke B. Wiewelhove T. Raeder C. Ferrauti A. Meyer T. Pfeiffer M. (2017). Evaluation of psychological measures for the assessment of recovery and stress during a shock-microcycle in strength and high-intensity interval training. *Perform. Enhanc. Health* 5 147–157. 10.1016/j.peh.2017.08.001

[B31] Hulin B. T. Gabbett T. J. Blanch P. Chapman P. Bailey D. Orchard J. W. (2014). Spikes in acute workload are associated with increased injury risk in elite cricket fast bowlers. *Br. J. Sports Med.* 48 708–712. 10.1136/bjsports-2013-092524 23962877

[B32] Hulin B. T. Gabbett T. J. Lawson D. W. Caputi P. Sampson J. A. (2016). The acute:chronic workload ratio predicts injury: high chronic workload may decrease injury risk in elite rugby league players. *Br. J. Sports Med.* 50 231–236. 10.1136/bjsports-2015-094817 26511006

[B33] Julian R. Meyer T. Fullagar H. H. K. Skorski S. Pfeiffer M. Kellmann M. (2017). Individual patterns in blood-borne indicators of fatigue-trait or chance. *J. Strength Cond. Res.* 31 608–619. 10.1519/JSC.0000000000001390 28212266

[B34] Kellmann M. (2000). Psychologische methoden der erholungs-beanspruchungs-diagnostik. [Psychological methods for the assessment of recovery and stress]. *Deutsche Z. Sportmed.* 51 253–258.

[B35] Kellmann M. (2002a). Psychologische erholungs- und beanspruchungssteuerung im ruder- und radsport. [Psychological monitoring of stress and recovery in rowing and cycling]. *Leistungssport* 32 23–26.

[B36] Kellmann M. (2002b). “Underrecovery and overtraining: different concepts-similar impact,” in *Enhancing Recovery: Preventing Underperformance in Athletes* ed. Kellmann M. (Champaign, IL: Human Kinetics) 3–24.

[B37] Kellmann M. (2010). Preventing overtraining in athletes in high-intensity sports and stress/recovery monitoring. *Scand. J. Med. Sci. Sports* 20(Suppl. 2) 95–102. 10.1111/j.1600-0838.2010.01192.x 20840567

[B38] Kellmann M. Beckmann J. (eds). (2018). *Sport, Recovery, and Performance: Interdisciplinary Insights.* New York, NY: Routledge.

[B39] Kellmann M. Bertollo M. Bosquet L. Brink M. S. Coutts A. Duffield R. (2018). Recovery and performance in sport: consensus statement. *Int. J. Sports Physiol. Perform.* 13 240–245. 10.1123/ijspp.2017-0759 29345524

[B40] Kellmann M. Kallus K. W. (2001). *Recovery Stress Questionnaire for Athletes: User Manual; [CD-ROM Included].* Champaign, IL: Human Kinetics.

[B41] Kellmann M. Kallus K. W. (2016). “Recovery-stress questionnaire for athletes,” in *The Recovery-Stress Questionnaires: User Manual* eds Kallus K. W. Kellmann M. (Frankfurt: Pearson) 86–134.

[B42] Kellmann M. Kölling S. Hitzschke B. (2016). *Das Akutmaß und die Kurzskala zur Erfassung von Erholung und Beanspruchung im Sport - Manual: [The Acute Measure and the Short Scale of Recovery and Stress for Sports - Manual]. Auflage, Stand* Vol. 1 Hellenthal: Sportverlag Strauß.

[B43] Kellmann M. Patrick T. Botterill C. Wilson C. (2002). “The recovery-cue and its use in applied settings: practical suggestions regarding assessment and monitoring of recovery,” in *Enhancing Recovery: Preventing Underperformance in Athletes* ed. Kellmann M. (Champaign, IL: Human Kinetics) 219–229.

[B44] Kenttä G. Hassmén P. (1998). Overtraining and recovery. A conceptual model. *Sports Med.* 26 1–16. 10.2165/00007256-199826010-00001 9739537

[B45] Kölling S. Hitzschke B. Holst T. Ferrauti A. Meyer T. Pfeiffer M. (2015). Validity of the acute recovery and stress scale. Training monitoring of the German junior national field hockey team. *Int. J. Sports Sci. Coach.* 10 529–542. 10.1260/1747-9541.10.2-3.529

[B46] Kölling S. Steinacker J. M. Endler S. Ferrauti A. Meyer T. Kellmann M. (2016). The longer the better. Sleep-wake patterns during preparation of the World Rowing Junior Championships. *Chronobiol. Int.* 33 73–84. 10.3109/07420528.2015.1118384 26730643

[B47] Lambert M. I. Mujika I. (2013). “Overtraining prevention,” in *Recovery for Performance in Sport* eds Hausswirth C. Mujika I. (Champaign, IL: Human Kinetics) 23–28.

[B48] Laux P. Krumm B. Diers M. Flor H. (2015). Recovery-stress balance and injury risk in professional football players. A prospective study. *J. Sports Sci.* 33 2140–2148. 10.1080/02640414.2015.1064538 26168148 PMC4673559

[B49] Mäestu J. Jürimäe J. Jürimäe T. (2005). Monitoring of performance and training in rowing. *Sports Med.* 35 597–617. 10.2165/00007256-200535070-00005 16026173

[B50] Maiwald M. Rogge K. (2005). “Zeitreihenanalyse von psychologischen und physiologischen variablen: eine langzeit-einzelfalluntersuchung. [Time series analysis of psychological and physiological variables: a long-term case-by-case study],” in *Zeitreihenanalysen: Mit Beispielen aus der Psychologie* ed. Werner J. (Berlin: Logos-Verl) 289–302.

[B51] Martin D. T. Andersen M. B. Gates W. (2000). Using Profile of Mood States (POMS) to monitor high-intensity training in cyclists: group versus case studies. *Sport Psychol.* 14 138–156. 10.1123/tsp.14.2.138

[B52] McNair D. Lorr M. Droppeleman L. F. (1971). *Manual for the Profile of Mood States* 1st Edn. San Diego, CA: Educational and Industrial Testing Service.

[B53] Meeusen R. Duclos M. Foster C. Fry A. Gleeson M. Nieman D. (2013). Prevention, diagnosis and treatment of the overtraining syndrome. Joint consensus statement of the European College of Sport Science (ECSS) and the American College of Sports Medicine (ACSM). *Eur. J. Sport Sci.* 13 1–24. 10.1080/17461391.2012.730061 23247672

[B54] Morgan W. P. Brown D. R. Raglin J. S. O’Connor P. J. Ellickson K. A. (1987). Psychological monitoring of overtraining and staleness. *Br. J. Sports Med.* 21 107–114. 10.1136/bjsm.21.3.107 3676635 PMC1478455

[B55] Mujika I. Busso T. Lacoste L. Barale F. Geyssant A. Chatard J. C. (1996). Modeled responses to training and taper in competitive swimmers. *Med. Sci. Sports Exerc.* 28 251–258. 10.1097/00005768-199602000-00015 8775162

[B56] Murray N. B. Gabbett T. J. Townshend A. D. Blanch P. (2017a). Calculating acute:chronic workload ratios using exponentially weighted moving averages provides a more sensitive indicator of injury likelihood than rolling averages. *Br. J. Sports Med.* 51 749–754. 10.1136/bjsports-2016-097152 28003238

[B57] Murray N. B. Gabbett T. J. Townshend A. D. Hulin B. T. McLellan C. P. (2017b). Individual and combined effects of acute and chronic running loads on injury risk in elite Australian footballers. *Scand. J. Med. Sci. Sports* 27 990–998. 10.1111/sms.12719 27418064

[B58] Nagle J. McMillan J. Munkasy B. Joyner B. Roorda A. Scott M. (2015). Changes in swim performance and perceived stress and recovery in female collegiate swimmers across a competitive season. *J. Swim. Res.* 23 44–53.

[B59] Nässi A. Ferrauti A. Meyer T. Pfeiffer M. Kellmann M. (2017). Development of two short measures for recovery and stress in sport. *Eur. J. Sport Sci.* 17 894–903. 10.1080/17461391.2017.1318180 28463598

[B60] Nielsen R. O. Parner E. T. Nohr E. A. Sorensen H. Lind M. Rasmussen S. (2014). Excessive progression in weekly running distance and risk of running-related injuries: an association which varies according to type of injury. *J. Orthop. Sports Phys. Ther.* 44 739–747. 10.2519/jospt.2014.5164 25155475

[B61] O’Connor P. J. Puetz T. W. (2005). Chronic physical activity and feelings of energy and fatigue. *Med. Sci. Sports Exerc.* 37 299–305. 10.1249/01.MSS.0000152802.89770.CF 15692327

[B62] Pelka M. Schneider P. Kellmann M. (2017). Development of pre- and post-match morning recovery-stress states during in-season weeks in elite youth football. *Sci. Med. Football* 34 1–6. 10.1080/24733938.2017.1384560

[B63] Raeder C. Wiewelhove T. Simola R. A. D. P. Kellmann M. Meyer T. Pfeiffer M. (2016). Assessment of fatigue and recovery in male and female athletes after 6 days of intensified strength training. *J. Strength Cond. Res.* 30 3412–3427. 10.1519/JSC.0000000000001427 27093538

[B64] Salminen S. Liukkonen J. Hanin Y. Hyvönen A. (1995). Anxiety and athletic performance of finnish athletes: application of the zone of optimal functioning model. *Pers. Individ. Dif.* 19 725–729. 10.1016/0191-8869(95)00096-O

[B65] Saw A. E. Kellmann M. Main L. C. Gastin P. B. (2017). Athlete self-report measures in research and practice: considerations for the discerning reader and fastidious practitioner. *Int. J. Sports Physiol. Perform.* 12 S2127–S2135. 10.1123/ijspp.2016-0395 27834546

[B66] Saw A. E. Main L. C. Gastin P. B. (2016). Monitoring the athlete training response: subjective self-reported measures trump commonly used objective measures: a systematic review. *Br. J. Sports Med.* 50 281–291. 10.1136/bjsports-2015-094758 26423706 PMC4789708

[B67] Schimpchen J. Wagner M. Ferrauti A. Kellmann M. Pfeiffer M. Meyer T. (2017). Can cold water immersion enhance recovery in elite Olympic weightlifters? An individualized perspective. *J. Strength Cond. Res.* 31 1569–1576. 10.1519/JSC.0000000000001591 28538307

[B68] Schmitz A. (2000). *“Erkennung von Nichtlinearitäten und Wechselseitigen Abhängigkeiten in Zeitreihen: [Detection of Nonlinearities and Interdependencies in Time Series].* Doctoral dissertation, Bergische Universität Wuppertal Wuppertal.

[B69] Schmitz B. (1996). “Dynamische interaktion in der trainingswissenschaft: multivariate ARIMA-modelle: [Dynamic interaction in exercise science: multivariate ARIMA models],” in *Proceedings of the Workshop Zeitreihenanalyse und “Multiple Statistische Verfahren” in der Trainingswissenschaft: 16 und 17 Juni 1995 in der Sportwissenschaftlichen Fakultät der Universität Leipzig und im Institut für Angewandte Trainingswissenschaft e.V* Vol. 1 ed. Krug J. (Cologne: Sport und Buch Strauss) 11–44.

[B70] Seiler K. S. Kjerland G. O. (2006). Quantifying training intensity distribution in elite endurance athletes: is there evidence for an “optimal” distribution? *Scand. J. Med. Sci. Sports* 16 49–56. 10.1111/j.1600-0838.2004.00418.x 16430681

[B71] Smith D. J. (2003). A framework for understanding the training process leading to elite performance. *Sports Med.* 33 1103–1126. 10.2165/00007256-200333150-00003 14719980

[B72] Soligard T. Schwellnus M. Alonso J.-M. Bahr R. Clarsen B. Dijkstra H. P. (2016). How much is too much? (Part 1) International Olympic Committee consensus statement on load in sport and risk of injury. *Br. J. Sports Med.* 50 1030–1041. 10.1136/bjsports-2016-096581 27535989

[B73] Toubekis A. G. Drosou E. Gourgoulis V. Thomaidis S. Douda H. Tokmakidis S. P. (2013). Competitive performance, training load and physiological responses during tapering in young swimmers. *J. Hum. Kinet.* 38 125–134. 10.2478/hukin-2013-0052 24233022 PMC3827763

[B74] Wallace L. Coutts A. Bell J. Simpson N. Slattery K. (2008). Using session-RPE to monitor training load in swimmers. *Strength Cond. J.* 30 72–76. 10.1519/SSC.0b013e31818eed5f

[B75] Wallace L. K. Slattery K. M. Coutts A. J. (2009). The ecological validity and application of the session-RPE method for quantifying training loads in swimming. *J. Strength Cond. Res.* 23 33–38. 10.1519/JSC.0b013e3181874512 19002069

[B76] Weinberg R. S. Gould D. (2007). *Foundations of Sport and Exercise Psychology* 4th Edn. Champaign, IL: Human Kinetics.

[B77] Wiewelhove T. Raeder C. Meyer T. Kellmann M. Pfeiffer M. Ferrauti A. (2016). Effect of repeated active recovery during a high-intensity interval-training shock microcycle on markers of fatigue. *Int. J. Sports Physiol. Perform.* 11 1060–1066. 10.1123/ijspp.2015-0494 26999645

[B78] Williams S. West S. Cross M. J. Stokes K. A. (2016). Better way to determine the acute:chronic workload ratio? *Br. J. Sports Med.* 51 209–210. 10.1136/bjsports-2016-096589 27650255

[B79] Ziemainz H. Peters S. (2010). Die Messung aktuellen wohlbefindens im gesundheitssport. Ein kritisches review [Measurement of actual well-being in wellness sports. A critical review]. *Sportwissenschaft* 40 174–181. 10.1007/s12662-010-0130-3

